# Fueling the Heart: What Are the Optimal Dietary Strategies in Heart Failure?

**DOI:** 10.3390/nu16183157

**Published:** 2024-09-18

**Authors:** Anahita Ataran, Alexander Pompian, Hamidreza Hajirezaei, Rehman Lodhi, Ali Javaheri

**Affiliations:** 1 Center for Cardiovascular Research, Washington University School of Medicine, St. Louis, MO 63110, USA; attaran@wustl.edu (A.A.); pompian@wustl.edu (A.P.); hajirezaei@wustl.edu (H.H.); lodhi@wustl.edu (R.L.); 2 John Cochran VA Hospital, St. Louis, MO 63110, USA

**Keywords:** heart failure, diet, calorie restriction, weight loss, ketogenic diet, Mediterranean-style diet

## Abstract

Objectives: Heart failure (HF) is a global health concern with rising incidence and poor prognosis. While the essential role of nutritional and dietary strategies in HF patients is acknowledged in the existing scientific guidelines and clinical practice, there are no comprehensive nutritional recommendations for optimal dietary management of HF. Methods: In this review, we discuss results from recent studies on the obesity paradox and the effects of calorie restriction and weight loss, intermittent fasting, the Western diet, the Mediterranean diet, the ketogenic diet, and the DASH diet on HF progression. Results: Many of these strategies remain under clinical and basic investigation for their safety and efficacy, and there is considerable heterogeneity in the observed response, presumably because of heterogeneity in the pathogenesis of different types of HF. In addition, while specific aspects of cardiac metabolism, such as changes in ketone body utilization, might underlie the effects of certain dietary strategies on the heart, there is a critical divide between supplement strategies (i.e., with ketones) and dietary strategies that impact ketogenesis. Conclusion: This review aims to highlight this gap by exploring emerging evidence supporting the importance of personalized dietary strategies in preventing progression and improving outcomes in the context of HF.

## 1. Epidemiology and Metabolic Alteration in Heart Failure

A simple definition of heart failure (HF) is that the heart pumps less efficiently, either with lower-than-normal output or under increased filling pressures. In the case of end-stage disease, left ventricular dysfunction leads to insufficient cardiac output, failure to supply the metabolic demands of the body, and volume overload with multi-organ congestion [1]. In developed countries, HF affects 1% to 3% of the general population [2], with a 5-year mortality rate of approximately 50% [3]. HF is the leading cause of hospitalization among older adults, with increasing prevalence in the elderly [4], ranging from 20 per 1000 in individuals aged 65 to 69 to over 80 per 1000 in those aged 85 and older [5,6]. Other major risk factors for HF include obesity, socioeconomic status, hypertension, coronary heart disease (CHD), diabetes, chronic kidney disease, smoking, chronic obstructive pulmonary disease (COPD), and rheumatic heart disease [1,6,7]. Additionally, HF can develop after myocarditis secondary to viral infections, including parvovirus, enteroviruses, adenovirus, coxsackievirus B, human herpesvirus 6, COVID-19, and others [8,9]. Although age is categorized as a non-modifiable risk factor, different studies have been conducted to explore methods aimed at decelerating the pace of cardiovascular aging [1,10,11]. In this context, cardiovascular aging encompasses a variety of biological changes, such as cellular senescence, telomere shortening, altered intercellular communication, chronic inflammation, and genomic instability [1,12]. The convergence of these conditions, coupled with the prevalent chief complaint of anorexia and malnutrition experienced by many HF patients, can disrupt metabolic homeostasis [13]. Consequently, as HF progresses to more advanced stages, patients can develop cachexia and sarcopenia, further exacerbating HF debility [14,15,16].

Metabolic disruption is central to the pathology of HF. Under normal conditions, myocardial tissue dynamically utilizes diverse substrates to fulfill its considerable energy demands and can generate adenosine triphosphate (ATP) via two primary means, namely glycolysis and mitochondrial oxidative phosphorylation. Of these, mitochondrial oxidative phosphorylation predominates in ATP production [17,18], with fatty acid oxidation contributing to 70–90% of ATP production [19]. In a healthy heart, metabolic flexibility allows for the seamless transition between different energy substrates, depending on factors such as substrate availability, cardiac workload, and hormonal status [20]. However, due to impaired crucial cellular processes, including impaired mitochondrial function and diminished biogenesis, metabolic adaptability is compromised in HF, and insufficient energy production can be a major underlying mechanism of cardiac dysfunction in heart failure [17,21].

Given these complex metabolic challenges, HF is classified into the following three main subtypes: (i) heart failure with reduced ejection fraction (HFrEF), defined as left ventricular EF < 40%; (ii) heart failure with preserved ejection fraction (HFpEF), with EF > 50%; and (iii) heart failure with mid-range ejection fraction (HFmrEF), with EF ranging between >40% and <50% [5,22,23,24,25], HFrEF and HFpEF are better studied than HFmrEF. Despite the distinct classifications of HF subtypes, there are still limited human clinical trials addressing the optimal dietary regimen for each specific subtype. Herein, we delve into different dietary interventions that mitigate or aggravate energy metabolism in cardiac tissue and how they could be tailored to the evolving stages of HF to improve the duration and quality of life and mitigate disease progression, noting that further research is essential to elucidate the most effective approaches.

## 2. Insights and Gaps in Dietary Approaches to Prevent and Treat Heart Failure

Although research continues to explore how modulating nutrient intake can mitigate metabolic imbalances associated with HF, there are currently no detailed dietary guidelines customized specifically for this condition [14]. Preventive dietary strategies in the context of HF mainly include early interventions to prevent the development of HF-associated risk factors, including hypertension, obesity, and the onset of disease [26]. To date, several dietary regimens have been shown to mitigate HF risk factors, with some even implicated as therapeutic with respect to pre-existing HF.

### 2.1. Dietary Approaches to Stop Hypertension (DASH) Diet

The Dietary Approaches to Stop Hypertension (DASH) diet is a well-established diet for preventing hypertension and consequent heart failure. The original diet, developed in the early 1990s, emphasized “fruits, vegetables, and low-fat dairy” and was found to be effective at lowering both systolic and diastolic blood pressure in the original clinical trial [27,28]. Following this, a second trial was carried out, testing the diet in combination with a varying sodium intake. In the trial, across three levels of sodium intake (high: ~3.3 g/2100 kcal; intermediate: ~2.5 g/2100 kcal; and low: ~1.5 g/2100 kcal), the DASH diet in tandem with lower sodium decreased blood pressure more than either variable individually [29]. Given the average American’s sodium intake (~3.5 g/day), DASH guidelines were updated to recommend 2300 mg sodium daily, noting that 1500 mg/day lowers blood pressure even more [30,31,32,33].

In the context of HF, increased sodium intake is associated with a 19% increase in incident HF (after adjusting for demographic and lifestyle variables) [34,35]. However, it should be noted that there are arguments advocating against sodium restriction alone in patients with HF. Especially at levels <1500 mg per day, patients may find adherence to sodium restriction “difficult or impossible” [36]. Additionally, animal studies have shown that extremely low-sodium diets can exacerbate HF through renin–angiotensin–aldosterone system (RAAS) activation, which causes salt and water retention to increase preload and afterload [37]. Thus, a lack of evidence makes it difficult to recommend a “lower limit” of sodium intake that patients should adopt to attenuate existing HF. Nonetheless, adherence to the standard DASH diet, as a whole, has been inversely associated with the incidence of heart failure, mainly by lowering blood pressure and increasing insulin sensitivity, especially among individuals less than 75 years old [38,39,40]. Additionally, although more sparse, data also suggest that adherence to the DASH diet is associated with a modest reduction in HF mortality [41]. Therefore, holistic implementation of the DASH diet can be reasonably recommended as a dietary strategy for HF patients. Although this diet has been clearly shown to improve cardiovascular disease risk factors, the pros and cons of long-term adherence to this diet should be further investigated [42,43].

### 2.2. Mediterranean Diet

Like the DASH diet, the Mediterranean diet emphasizes plant-derived foods (fruits, vegetables, nuts, and legumes), whole grains, and a moderate intake of fish/poultry and is generally low in saturated fats, red meat, alcohol, and processed foods [44,45,46,47]. However, it should be noted that moderate consumption of wine (preferably red wine) with meals has also been suggested as an essential element of the Mediterranean diet [47]. In contrast to the DASH diet, which limits total fat (especially saturated fatty acids), the Mediterranean diet suggests increasing unsaturated fatty acid intake, especially though the consumption of olive oil [35]. Thus diet has been shown to reduce the risk of cardiovascular diseases, including HF, in both healthy individuals and people with prior cardiovascular diseases [48]. A review by Oppedisano et al. highlighted that dietary supplementation with *n*-3 polyunsaturated fatty acid (PUFAs) has anti-inflammatory and cardioprotective effects, with benefits linked to dosage and treatment duration [49]. However, the exact underlying mechanisms of the protective effects of this dietary regimen have yet to be determined [44]. Olive oil/unsaturated fat consumption (especially as a substitute for saturated fats) has been associated with decreased risk of cardiovascular disease and all-cause mortality, although HF-specific trials have yet to be carried out [50,51,52]. Importantly, like the DASH diet, no singular aspect of the Mediterranean diet has been found to be as beneficial as the overall diet [46]. A diet similar to the Mediterranean diet is the plant-based EAT-Lancet diet, although it places a greater emphasis on legumes and cereals [53]. This diet has been found to lower the risk of heart failure with higher adherence, identifying eight plasma proteins that may mediate this relationship (Apolipoprotein-M, Growth Differentiation Factor-15, Interleukin-6, TIM, Cathepsin D, CCL20, FS, and FUR). A study by Zhang, et al. proposed that these plasma proteins are inversely associated with the risk of HF and might mediate the protective effects of this diet [54].

## 3. Weight Loss Strategies and Heart Failure

Obesity, which is defined as a body mass index (BMI) of 30 kg/m^2^ or higher, is a well-known risk factor for HF [55,56]. For example, in patients with type 2 diabetes, weight loss (average of 15%) as a result of glucagon-like peptide 1 receptor agonists (GLP-1 RAs) was found to lower the risk of HF hospitalization by 9% (HR: 0.91, 95% CI: 0.83–0.99; *p* = 0.028) according to a meta-analysis of seven randomized controlled trials [57]. Moreover, an analysis of HF incidence following bariatric surgery (15–25% weight loss) found the incidence to be reduced by half (HR: 0.50, 95% CI 0.38–0.66, *p* ≤ 0.001) [58]. Interestingly, however, once individuals have developed HF, a higher BMI is associated with improved outcomes [59,60,61,62]. This “obesity paradox” raises an important conundrum, specifically how obesity should be treated clinically in the context of HF. Although more evidence is needed, current recommendations do not purport GLP-1 RAs as protective in overweight/obese patients (BMI > 25 kg/m^2^) with pre-existing advanced HFrEF; GLP-1 RA treatment may increase the risk of HF rehospitalization (HR 1.33, 95% CI 0.83–2.12) [63], although evolving evidence suggests GLP-1 RA could certainly benefit those with HFpEF [64]. On the other hand, HF patients with a BMI > 35 kg/m^2^ who underwent bariatric surgery experienced a lower risk of all-cause mortality (HR, 0.55 [95% CI, 0.49–0.63]; *p* < 0.001) and a greater reduction in HF hospitalization (rate ratio, 0.72 [95% CI, 0.67–0.77]; *p* < 0.001) [65]. Therefore, further investigation into the merit of weight loss-oriented dietary strategies in the context of HF is warranted.

### Intermittent Fasting and Caloric Restriction

Various dietary approaches, such as the 16/8 method, the 5:2 diet, and the 12/12 method, have been developed to address the cardiometabolic benefits and drawbacks of intermittent fasting (IF) [66]. Various clinical trials have also explored the effects of fasting on body weight, insulin sensitivity, and overall metabolic health [66,67,68]. A recent human clinical trial of patients with ST-elevation myocardial infarction (STEMI) showed that IF significantly improved the left ventricular ejection fraction and reduced diastolic blood pressure compared to a regular diet, with improvement occurring up to 6 months after STEMI [69]. However, to date, no randomized controlled trials in humans have specifically investigated the effects of fasting on HF progression. In contrast, animal studies suggested that alternate-day fasting (ADF), a commonly used form IF in rodent models, potentiates cardiotoxic effects of doxorubicin chemotherapy, which causes HF in humans, highlighting the need for cautious exploration of IF in this context [70]. Mechanistically, ADF increases myocardial nuclear transcription factor EB (TFEB), which drives HF progression after doxorubicin [70]. However, other rodent studies corroborate the potential beneficial effects of IF in the cardiometabolic disease setting, suggesting that the role of IF may be context-dependent [71,72,73]. Numerous other studies have reviewed both continuous energy restriction and intermittent energy restriction for their other effects in terms of reducing body fat mass, promoting weight loss, and improving cardiovascular health [74,75,76]. However, only two randomized clinical trials have been conducted to investigate caloric restriction in heart failure (Table 1). First, in a randomized controlled trial involving geriatric obese patients with HFpEF, caloric restriction alone led to significant reductions in serum interleukin-6, TNF-α-receptor-I, growth differentiation factor-15, cystatin C, and *N*-terminal pro-b-type natriuretic peptide (Table 1), resulting in improved physical performance and exercise tolerance compared to the group that combined caloric restriction with aerobic exercise [77]. In a separate randomized controlled trial, no significant impact on patients’ quality of life was found when they underwent caloric restriction, aerobic exercise, or a combination of both, as assessed through a questionnaire-based evaluation (Table 1) [78]. On the other hand, insufficient caloric intake is known to worsen post-discharge quality of life and increase the burden of readmission in HF patients [79]. Therefore, the potential benefits of calorie restriction must be determined on a case-by-case basis [76]. Indeed, it is possible that the success or failure of these strategies might be related to the underlying HF syndrome (HFpEF vs. HFrEF subtypes) or comorbidities such as obesity. Although a sustained 5–10% weight loss is recommended in HF patients with a BMI > 35 kg/m^2^, weight loss puts lower-BMI groups at greater risk of mortality, which is likely reflective of cachexia [80]. Without the addition of resistance training, weight lost across all groups can be the result of loss of skeletal muscle mass [81]. Thus, calorie restriction alone cannot be universally recommended as an HF treatment but as a strategy to mitigate one of its main risk factors, namely obesity [80].

## 4. Alternative Dietary Strategies for Heart Failure

### High-Fat, Western, and Ketogenic Diets

In Western nations, the most common diets are high in calories, fats, and sugars such as fructose and sucrose, contributing to chronic conditions such as hypertension and type 2 diabetes, which are other common risk factors for HF [82,83,84]. Generally, high-fat and Western-type diets are used to induce obesity, metabolic syndrome, and diabetes in mouse models and are associated with cardiac dysfunction [85,86,87,88]. In such models, long-term adherence to the Western diet specifically leads to impaired glucose tolerance and changes in lipid storage dynamics (higher triglyceride levels with reduced turnover), resulting in the development of a unique phenotype of metabolic stress.

While a prior study suggested that a high-fat diet (60% fat, 20% carbs, and 20% protein, compared to a 17% fat, 54% carb, 29% protein chow diet) causes systolic dysfunction in mice by reducing sirtuin 3 [87]; however, these results have been called into question by other models that suggest a high-fat diet can improve cardiac function [85,86]. Interestingly, in a model of mitochondrial pyruvate carrier deficiency that caused reduced EF in mice, high-fat diet feeding with either a ketogenic or non-ketogenic diet reversed cardiac dysfunction [89]. Ketones may have protective effects on cardiac metabolism due to their effective energy production compared to FAs [90]. In essence, a ketogenic diet—high in fat and low in carbohydrates—aims to induce ketosis by decreasing carbohydrates, leading to increased myocardial fatty acid oxidation and ketone utilization. Given that ketones are considered an efficient and increasingly utilized source of fuel in a failing heart [91], the ketogenic diet, along with beta-hydroxybutyrate (BHB, a key byproduct of ketosis) supplementation, has been increasingly studied in recent years [92,93,94,95,96]. One mechanism by which the ketogenic diet may improve myocardial health is through BHB-mediated inhibition of the NLRP3 inflammasome and consequent improvement of chronic inflammation [97]. However, in the context of HF, animal studies incorporating a ketogenic diet have yielded mixed results, as recent studies suggest that such a diet induces cellular senescence in multiple organs, including the heart [98,99]. Additionally, in humans, how the ketogenic diet is implemented may influence the extent of its therapeutic effects. For example, carbohydrate-restricted diets are often associated with increased mortality—but only when carbs are replaced with animal-derived protein and fat. When replaced with equivalent plant-based counterparts, Seidelmann et al. found that mortality decreased [100]. In comparison with a high-carbohydrate diet, a low-carbohydrate diet has been shown to lead to greater weight loss due to the enhanced loss of water weight and fat loss, with beneficial effects on glycemic control [101]. Additionally, one model comparing a continuous ketogenic diet and an alternate-day ketogenic diet showed that the continued ketogenic diet failed to protect against HF, whereas the alternate-day ketogenic diet exerted significant cardioprotective effects against HF and did not impair the capacity of hepatic ketogenesis [102]. Thus, the evidence suggests that our understanding of the mechanisms behind the ketogenic diet and its role in treating HF requires further study.

## 5. Supplemental Strategies in Heart Failure

Alongside dietary and lifestyle modifications for heart failure patients, considerable investment and interest have been demonstrated in supplements (nutriceuticals) to enhance heart failure management. For example, a clinical trial testing BHB supplementation in 24 HFrEF patients showed significant increases in cardiac output, stroke volume, and LVEF vs. placebo. The patients underwent 11C-Acetate PET examinations to assess myocardial energy expenditure (MEE) and myocardial oxygen consumption (MVO2), and it was found that BHB infusion led to increases in MVO2, while MEE remained the same, suggesting that BHB may improve cardiac function without impairing the energy expenditure of the heart. Further clinical trials are currently underway [103,104,105,106]. Beetroot juice is also under investigation, having been shown to reduce infarct size and improve heart function in mice, in addition to its antihypertensive properties and ability to increase exercise capacity in humans with HFpEF [107,108]. Mechanistically, inorganic nitrate/nitrite content in beetroot is thought to be responsible for its ameliorative effects; however, randomized trials on nitrites have yet to show a benefit [109]. Ongoing randomized clinical trials have yet to be concluded on inorganic nitrates [110]. In HF patients with vitamin deficiency, vitamin supplementation can also exert significant therapeutic effects. For example, vitamin B1 (thiamine) deficiency is known to cause HF, and its repletion has been shown to significantly improve LVEF in B1-deficient patients with HFrEF, along with improvements in the 6 min walking test [111,112,113]. Vitamin C deficiency is also common in HF patients, and relative plasma levels thereof can predict the risk of HF across men and women aged 39–79. Although there has been investigation into several other supplementation strategies in HF (e.g., taurine, vitamin D, etc.), treating various nutrient deficiencies with supplementation can be avoided altogether by adhering to dietary strategies like the aforementioned Mediterranean and DASH diets [114].

## 6. Conclusions and Future Perspectives

Taken together, while some popular dietary strategies offer limited benefits and require further research, one principle remains consistent, namely that dietary strategies in patients with HF are most effective when aligned with the principles of the DASH or Mediterranean diets, which emphasize plant-based foods and low saturated fats [98]. For patients who may benefit from sodium restriction, the DASH diet, which implements many aspects of the Mediterranean diet, also shows promise.

It should be noted, as previously stated, that a change in one’s dietary pattern does not unilaterally prevent HF but, rather, mitigates its risk factors, such as obesity and hypotension [34,46]. Thus, lifestyle changes with the goal of preventing HF should target these risk factors rather than one specific dietary pattern. In addition to traditional dietary approaches, further research should also explore the role of personalized nutrition. Given individual differences in genetics, lifestyle, and metabolic responses, personalized nutrition has the potential to tailor dietary recommendations more effectively. A significant challenge in dietary studies is understanding the metabolic changes and adaptations that occur with different diets. Assessing the physiological response to various nutrient signaling pathways in a single organ is complex and may seem unrealistic. However, elucidating the underlying mechanisms of different nutrients could significantly impact the identification of the most effective diet for conditions such as heart failure. Looking ahead, more basic research and randomized trials of various scales and durations are needed to enhance our understanding of metabolic shifts under different diets and evaluate the long-term outcomes of different dietary regimens.

## 7. Limitations of the Study

One major limitation for dietary intervention studies, in general, is the sex-dependent differences in cardiometabolic pathophysiology, disease susceptibility, clinical presentation, prognosis, and treatment response. There is a major gap in the literature pointing dietary interventions in a sex-dependent manner. Another main limitation of dietary studies is the lack of evidence addressing dietary interventions specifically targeting HFpEF vs. HFrEF. Furthermore, evidence supporting diet implementation according to heart failure stage is currently limited. Our recommendation highlights the potential importance of tailoring dietary interventions to the specific needs of patients at different stages of heart failure, even though existing research is sparse. We believe that further studies are necessary to explore and validate stage-specific dietary approaches, which could potentially improve patient outcomes in heart failure management.

## Figures and Tables

**Table 1:** Comparison of two clinical randomized trials using caloric restriction interventions in patients with HF.

Parameter/Study	Justice et al. [77]	Kitzman et al. [78]
Target group	Obese HFpEF	Obese HFpEF
Intervention	CR * vs. CR and/or EX **	CR vs. CR and/or EX
Intervention duration	20 weeks	20 weeks
Number of participants	88	100
Age (years), mean ± SD	66.6 ± 5.3	67 ± 5
Sex	81% female	81% female
BMI in kg/m^2^, mean ± SD	39.3 ± 6.3	39.3 ± 5.6
Biomarker index improvement with CR	−0.82 ± 0.58 points, *p* = 0.05	Not applicable
Biomarker index improvement with EX	−0.28 ± 0.59 points, *p* = 0.50	Not applicable

* Caloric restriction. ** Aerobic exercise training.
